# Construction and evaluation of a neonatal septic shock prediction model based on multimodal data

**DOI:** 10.1097/MD.0000000000042746

**Published:** 2025-06-06

**Authors:** Fan Liu, Jinlan Chen, Qinglan Huang, Xiaoning Yan, Zhen Li, Zheng Yan

**Affiliations:** a Department of Pediatrics/Neonatology, FuZhou First General Hospital Affiliated with Fujian Medical University, Fuzhou, Fujian, China.

**Keywords:** neonate, sepsis, septic shock, systemic inflammatory response syndrome

## Abstract

This study aimed to develop and validate a predictive model using multimodal data for early identification of septic shock in neonates with systemic inflammatory response syndrome (SIRS) or sepsis. This retrospective cohort study included neonates diagnosed with SIRS, sepsis, or septic shock at Fuzhou First General Hospital between January 2021 and December 2023. Univariate and multivariate logistic regression analyses were performed to identify independent predictors, and receiver operating characteristic curves were used to evaluate the model’s predictive performance. Multivariate analysis identified 7 independent predictors of septic shock, namely, the age of neonatal (OR = 2.293, 95% CI = 2.129–3.482), critical illness score (OR = 1.835, 95% CI = 1.582–2.354), cerebral oxygen saturation (ScO_2_) (OR = 0.289, 95% CI = 0.281–0.359), pulsatility index of right middle cerebral artery (OR = 0.837, 95% CI = 0.828–1.022), peak velocity (PSV) of left renal hilum (OR = 0.952, 95% CI = 0.868–1.157), procalcitonin (OR = 1.875, 95% CI = 1.725–2.061), and lactate (OR = 9.654, 95% CI = 8.612–10.572) were independent influencing factors for the occurrence of septic shock. Based on the result of the regression analysis, we constructed a model for predicting septic shock, namely, multimodal model = (−0.378 × neonatal age) − (0.145 × critical illness score) − (0.366 × ScO_2_) + (0.416 × pulsatility index of right middle cerebral artery) + (0.825 × PSV of left renal hilum) + (12.288 × procalcitonin) + (5.167 × lactate) + 1.804. And results of receiver operating characteristic curve showed that the area under curve of the multimodal model for predicting septic shock in the SIRS + septic shock population, sepsis + septic shock population, and SIRS + sepsis + septic shock population is 0.862, 0.746, and 0.820, respectively. A multimodal prediction model incorporating clinical, hemodynamic, and biochemical parameters demonstrated robust performance in early identification of neonatal septic shock. Further validation through multicenter studies is warranted.

## 1. Introduction

Neonates are particularly vulnerable to infections due to immature immune systems, with outcomes ranging from systemic inflammatory response syndrome (SIRS) to life-threatening septic shock.^[1,2]^ Sepsis, defined as organ dysfunction secondary to a dysregulated host response to infection, progresses to septic shock in severe cases, characterized by profound circulatory and metabolic derangements associated with high mortality rates.^[3]^ Early recognition of septic shock is critical for initiating timely resuscitation, yet current diagnostic tools – clinical signs, blood cultures, and isolated biomarkers – lack sensitivity and specificity.^[4,5]^ Therefore, it is important to predict the progression of neonatal infection in a timely manner to accurately identify neonates with septic shock.

At present, clinicians mainly rely on clinical feature recognition, blood cultures, and laboratory indicators to diagnose neonatal septic shock, but these diagnostic tools have many limitations and are affected by confounding factors.^[6,7]^ In addition, sepsis is a systemic disease that affects various aspects of circulation, including the myocardium, pulmonary blood vessels, systemic blood vessels, and microcirculation, so there is no universally accepted indicator as the only parameter to guide shock recovery in sepsis.^[8–10]^ Multimodal approaches integrating hemodynamic, metabolic, and microcirculatory parameters may enhance predictive accuracy. Therefore, it is necessary to use a combination of multimodal data to improve sensitivity and specificity for diagnosing septic shock.

In this retrospective study, we incorporate methods and concepts including physical examination, laboratory tests, imaging tests, and monitoring of various physiological data to integrate macroscopic hemodynamics, metabolic status, peripheral status, local and microcirculatory perfusion parameters to construct a multimodal model that can predict the occurrence of septic shock.

## 2. Patients and methods

### 2.1. Patients and study design

The retrospectively study enrolled 156 neonates admitted to the neonatal intensive care unit at Fuzhou First General Hospital between 2021 and 2023. And these newborns were eventually diagnosed with SIRS, sepsis or septic shock according The Third International Consensus Definitions for Sepsis and Septic Shock (Sepsis-3).^[3]^ The present study followed the Helsinki Declaration, and the Ethics Committee of FuZhou First General Hospital approved the study.

Inclusion criteria: postnatal age ≤ 28 days. Admitted to our hospital within 24 hours after diagnosis of SIRS or sepsis or onset of symptoms. No anti-shock or blood products treatment prior to the test of laboratory index. completion of diagnostic workup within 12 hours of admission. Exclusion criteria: congenital heart disease, congenital malformations, chromosomal disease or suspected inherited metabolic disorder. The mother of the neonatal patient has a history of drug addiction or has a disease that can be transmitted from mother to child. Hospitalization for <24 hours. No final diagnosis of illness in our hospital. Incomplete clinical data.

### 2.2. Data collection

According to medical records, we collect the following data from neonatal patients: Clinical data of newborn mothers, including age, gestation diabetes, number of pregnancies, number of deliveries, abort, perioperative infection, preventive antibiotics and gestational age. Baseline clinical data of neonatal patients upon admission, including the age, birth weight, fetal distress, first-minute Apgar score, fifth-minute Apgar, 10th-minute Apgar, respiratory rate, systolic blood pressure, diastolic blood pressure, sensitive pupil response, urine output, intrauterine infection, dopamine, critical illness score gender, amniotic fluid turbidity and heart rate. Imaging indexes upon admission, including transcutaneous oxygen pressure (PtcO_2_), transcutaneous carbon dioxide pressure (PtcCO_2_), perfusion index, cerebral oxygen saturation (ScO_2_), regional oxygen saturation (rSO_2_), peak velocity (PSV) of left middle cerebral artery (LMCA), perfusion index (PI) of LMCA, PSV of right middle cerebral artery (RMCA), PI of RMCA, PSV of left renal hilum (LRH), resistance index (RI) of (LRH), PSV of right renal hilum (RRH), RI of RRH and ejection fraction of the left ventricle (EF). Noninvasive hemodynamics indexes, including ventricular stroke volume (SV), cardiac stroke index, cardiac output (CO), CO index (CI), SV variation, flow time corrected, systemic vascular resistance (SVR), thoracic fluid conductivity, systemic vascular resistance, systemic vascular resistance index, index of myocardial contractility (ICON), shrink time ratio, pre-ejection phase, left ventricular ejection time, variation of myocardial contractility index (VIC), oxygen delivery (DO_2_) and mean arterial pressure. First laboratory indicator, including white blood cell count (WBC), Neutrophils, C-reactive protein, interleukin-6, procalcitonin, creatine kinase isoenzyme MB (CK-MB), N-terminal B-type natriuretic peptide precursor (NT-proBNP), lactate, urea and creatinine.

### 2.3. Statistical analysis

SPSS 20.0 software (IBM, Chicago) was used for statistical analysis in the present study. Qualitative data were presented as percentages, and the chi-square test or Fisher exact probability method was used to compare the differences of qualitative data between groups. Continuous data that conform to a normal distribution were compared using 1-way ANOVA with Bonferroni posttest between groups. And non-normally distributed continuous data were presented as median (interquartile range), 1-way ANOVA and Mann-Whitney U method to compare differences between groups. The variance inflation factor (VIF) performed a collinearity test on the study data, and the VIF ≥ 10 indicated that there was collinearity between the study indicators, and we solved the collinearity by reducing the correlation between the variables by centralization or standard deviation of the variables. Multivariate logistics regression analysis was used to identify independent influencing factors for the development of septic shock. The predictive value of the predictive model for the occurrence of septic shock was assessed using the receiver operating characteristic curve (ROC). *P* < .05 indicated that the difference was statistically significant.

### 2.4. Sample size determination

During sample size calculation for this multivariable prognostic model, we used the rule of thumb method of 10 events per parameter.^[11,12]^ According to previous research results,^[13]^ 6 factors were identified as independent risk factors for septic shock in neonatal sepsis. Subsequently, we selected data from 2021 to 2023 for investigation and determined that the incidence of septic shock in neonatal sepsis was 48.78%. In summary, the minimum sample size should be (6 × 10)/0.4878 = 123.

## 3. Results

### 3.1. Clinical data of newborn mothers

Of 156 neonates, 74 had SIRS, 42 sepsis, and 40 septic shock. Maternal gestational age, abortion history, and perioperative infections differed significantly among groups (*P* < .05). Neonates with septic shock exhibited lower Apgar scores, higher lactate, and altered cerebral/renal perfusion indices (*P* < .05). First, we compared the general clinical data of these newborn mothers, and found that there was no significant difference between these 3 groups on the age, gestation diabetes, number of pregnancies and number of deliveries (*P* > .05), while there was significant difference between these 3 groups on the abort, perioperative infection, preventive antibiotics and gestational age (*P* < .05). Details are presented in Table 1.

**Table 1:** Comparison of clinical data of newborn mothers.

Index	Septic shock group (n = 40)	Sepsis group (n = 42)	SIRS group (n = 74)	*F*/χ2	*P*-value
Age (yr)	30.73 ± 4.52	30.55 ± 4.32*	29.97 ± 4.56*	0.438	.646
Abort, n (%)	17 (42.50)	2 (4.76)**	2 (2.70)**	39.035	<.001
Gestational diabetes, n (%)	13 (32.50)	8 (19.05)*	14 (18.92)*	3.131	.209
Perioperative infection, n (%)	6 (15.00)	2 (4.76)*	2 (2.70)**	6.805	.033
Preventive antibiotics, n (%)	6 (15.00)	1 (2.38)**	3 (4.05)**	6.741	.034
No. of pregnancies (n)	2.50 ± 1.36	2.41 ± 1.40*	2.20 ± 1.32*	0.707	.495
No. of deliveries (n)	1.73 ± 0.78	1.76 ± 0.86*	1.62 ± 0.70*	0.486	.616
Gestational age (wk)	36.46 ± 3.71	38.47 ± 2.23**	37.66 ± 2.37**	5.510	.005

Continuous data that conform to a normal distribution are denoted as (mean ± standard deviation).

* Compared with septic shock group, *P* > .05.

** Compared with septic shock group, *P* < .05.

### 3.2. Baseline clinical data of neonatal patients upon admission

Significant differences were observed in baseline clinical characteristics among neonates in the systemic inflammatory response syndrome (SIRS), sepsis, and septic shock groups, including age, birth weight, fetal distress, first-minute Apgar score, fifth-minute Apgar score, 10th-minute Apgar score, respiratory rate, systolic blood pressure, diastolic blood pressure, urine output, serum procalcitonin ratio, intrauterine infection, dopamine administration, and critical illness score (all *P* < .05). No significant differences were detected in sex, amniotic fluid turbidity, or heart rate (*P* > .05). Details are presented in Table 2.

**Table 2:** Comparison of baseline clinical characteristics in neonatal patients.

Index	Septic shock group (n = 40)	Sepsis group (n = 42)	SIRS group (n = 74)	*Z*/*F*/χ^2^	*P*-value
Age (h)	1.39 ± 1.94	22.14 ± 26.21*	36.49 ± 53.44*	10.382	<.001
Male, n (%)	22 (55.00)**	23 (54.76)**	34 (45.95)**	1.242	.537
Birth weight (kg)	2.58 ± 0.66	3.09 ± 0.57*	3.01 ± 0.68*	7.665	<.001
Fetal distress, n (%)	18 (45.00)	6 (14.29)*	10 (13.51)*	17.004	<.001
AFT, n (%)	12 (30.00)	10 (23.81)**	12 (16.22)**	3.031	.220
First minute Apgar	6.58 ± 2.88	8.76 ± 0.79*	8.38 ± 1.28*	18.952	<.001
Fifth minute Apgar	8.03 ± 2.15	9.67 ± 0.57*	9.47 ± 1.02*	19.654	<.001
10th minute Apgar	8.58 ± 1.78	9.71 ± 0.46*	9.50 ± 1.37*	8.857	<.001
Respiratory rate (n)	66.00 ± 11.54	58.64 ± 7.68*	58.11 ± 8.73*	10.283	<.001
Heart rate (n)	147.13 ± 19.24	145.69 ± 17.19	145.65 ± 18.43	0.095	.910
SBP (mm Hg)	71.08 ± 13.32	78.12 ± 14.88*	80.07 ± 9.98*	7.051	.001
DBP (mm Hg)	37.68 ± 10.92	45.29 ± 8.75*	45.54 ± 9.41*	9.652	<.001
Urine output (mL)	70.97 ± 62.65	100.24 ± 65.16*	109.77 ± 68.92*	4.494	.013
SPR, n (%)	32 (80.00)	1 (2.38)*	1 (1.35)*	106.937	<.001
Intrauterine infection, n (%)	3 (7.50)	12 (28.57)*	9 (12.16)**	8.111	.017
Dopamine, n (%)	33 (82.50)	7 (16.67)*	10 (13.51)*	62.986	<.001
Critical illness score	98.0 (94.5, 102.0)	106.0 (102.0, 106)*	106.0 (99.5, 106)*	14.075	<.001

Continuous data follows a normal distribution with (mean ± standard deviation), otherwise represented as median (interquartile range).

AFT = amniotic fluid turbidity, SBP = systolic blood pressure, DBP = diastolic blood pressure, SPR = sensitive pupil response.

* Compared with Septic shock group, *P* < .05

** Compared with Septic shock group, *P* > .05.

### 3.3. Imaging related indexes in neonatal patients upon admission

All neonates underwent noninvasive imaging examinations at admission. Comparative analysis revealed significant differences in transcutaneous oxygen tension PtcO_2_, PtcCO_2_, ScO_2_, rSo_2_, PI of LMCA, PI of RMCA and PSV of LRH (*P* < .05), RI of LRH these 3 groups, while no significant difference on PtcCO_2_, perfusion, PSV of LMCA, PSV of RMCA, PSV of RRH, RI of RRH and EF (*P* > .05). Complete results are summarized in Table 3.

**Table 3:** Comparison of imaging indexes in neonatal patients.

Index	Septic shock group (n = 40)	Sepsis group (n = 42)	SIRS group (n = 74)	*F*/χ^2^	*P*-value
PtcO_2_ (mm Hg)	109.41 ± 47.15	91.71 ± 25.34*	93.48 ± 28.19*	3.690	.027
PtcCO_2_ (mm Hg)	33.95 (30.03, 40.45)	36.15 (30.88, 41.93)**	38.15 (32.83, 45.65)**	0.476	.622
Perfusion index (%)	0.97 ± 0.44	1.12 ± 0.54**	1.13 ± 0.51**	1.404	.249
ScO_2_ (%)	72.50 (67.25, 83.75)	79.00 (76.00, 83.25)*	82.00 (78.00, 85.00)*	9.032	<.001
rSO_2_ (%)	73.88 ± 7.22	81.50 ± 8.24*	81.68 ± 6.66*	16.926	<.001
PSV of LMCA (cm/s)	43.11 ± 13.81	42.62 ± 16.84**	45.01 ± 13.22**	0.447	.640
PI of LMCA	1.31 ± 0.30	1.20 ± 1.44**	1.16 ± 0.25**	2.917	.057
PSV of RMCA (cm/s)	40.84 ± 13.25	43.13 ± 16.06**	45.36 ± 13.31**	1.369	.257
PI of RMCA	1.35 (1.08, 1.65)	1.16 (0.93, 1.37)*	1.16 (1.02, 1.31)*	5.913	.003
PSV of LRH (cm/s)	48.53 (39.70, 64.00)	40.35 (36.20, 56.73)*	36.75 (30.95, 47.80)*	4.467	.013
RI of LRH	1.02 ± 0.45	0.70 ± 0.09*	0.71 ± 0.10*	24.413	<.001
PSV of RRH (cm/s)	40.92 (36.70, 53.70)	38.75 (31.93, 46.55)**	36.60 (30.40, 45.85)**	0.913	.403
RI of RRH	0.74 ± 0.10	0.72 ± 0.08**	0.72 ± 0.11**	0.434	.649
EF (%)	67.07 ± 6.14	68.09 ± 4.76**	69.04 ± 5.96**	1.568	.212

Continuous data follows a normal distribution with (mean ± standard deviation), otherwise represented as median (interquartile range).

EF = ejection fraction of the left ventricle, LMCA = left middle cerebral artery, LRH = left renal hilum, PI = perfusion index, PtcO_2_ = transcutaneous oxygen pressure, PtcCO_2_ = transcutaneous carbon dioxide pressure, PSV = peak velocity, RMCA = right middle cerebral artery, RI = resistance index, RRH = right renal hilum, ScO_2_ = cerebral oxygen saturation, rSO_2_ = regional oxygen saturation.

* Compared with septic shock group, *P* < .05.

** Compared with septic shock group, *P* > .05.

### 3.4. Noninvasive hemodynamics indexes in neonatal patients

Noninvasive hemodynamic monitoring was used to assess CO. Among the parameters evaluated, only the stroke index demonstrated significant differences across the 3 groups (*P* < .05). Details are presented in Table 4.

**Table 4:** Comparison of noninvasive hemodynamics indexes in neonatal patients.

Index	Septic shock group (n = 40)	Sepsis group (n = 42)	SIRS group (n = 74)	*F*/χ^2^	*P*-value
SV (mL)	5.88 (4.26, 7.54)	5.51 (4.76, 6.45)*	5.48 (4.52, 6.73)*	0.133	.716
SI (mL/m^2^)	33.00 (26.50, 42.25)	30.00 (25.00, 35.00)*	29.00 (24.50, 34.25)**	3.971	.021
CO (L/min)	0.80 (0.61, 1.11)	0.77 (0.62, 0.91)*	0.76 (0.56, 0.88)*	1.319	.253
CI (L/min/m^2^)	4.23 (3.52, 5.12)	3.90 (3.40, 4.33)*	3.90 (3.20, 4.40)*	0.879	.350
SVV (%)	24.85 ± 11.46	24.29 ± 9.63*	23.16 ± 13.75*	0.498	.481
FTc (ms)	278.03 ± 32.51	271.29 ± 28.68*	267.16 ± 31.94*	3.138	.078
TFC (kΩ)	68.76 ± 18.78	65.92 ± 17.66*	63.62 ± 21.17*	1.780	.184
SVR (dyn·s·cm^-5^)	7230.35 ± 4239.86	7471.26 ± 7191.52*	7594.75 ± 3677.23*	0.139	.710
SVRI (dyn·s·cm^-5^·m^2^)	1317.00 ± 476.74	1409.33 ± 1354.64*	1910.50 ± 3301.21*	1.591	.209
ICON (mW)	87.65 (61.80, 127.08)	75.60 (67.96, 95.75)*	75.25 (61.80, 97.53)*	0.550	.459
STR (kΩ)	0.51 (0.38, 0.66)	0.45 (0.43, 0.56)*	0.48 (0.43, 0.58)*	0.018	.895
PEP (ms)	84.00 (77.25, 90.75)	85.00 (80.25, 92.25)*	90.00 (81.00, 99.00)*	3.263	.073
LVET (ms)	185.35 ± 39.93	183.79 ± 34.38*	181.62 ± 33.35*	0.288	.592
VIC (L/1000 ms)	32.45 ± 16.47	30.55 ± 15.38*	28.65 ± 16.54*	1.426	.234
DO_2_ (mL/min)	197.0 (140.2, 251.0)	164.5 (123.7, 199.5)*	159.0 (120.5, 193.5)*	2.204	.114
MAP (mm Hg)	56.88 ± 9.45	56.76 ± 8.17*	57.88 ± 7.90*	0.371	.543

Continuous data follows a normal distribution with (mean ± standard deviation), otherwise represented as median (interquartile range).

CI = cardiac output index, CO = cardiac output, DO_2_ = oxygen delivery, FTc = flow time corrected, ICON = Index of myocardial contractility, LVET = left ventricular ejection time, MAP = mean arterial pressure, PEP = pre-ejection phase, SI = cardiac stroke index, STR = shrink time ratio, SV = ventricular stroke volume, SVR = systemic vascular resistance, SVRI = systemic vascular resistance index, SVV = stroke volume variation, TFC = thoracic fluid conductivity, VIC = variation of myocardial contractility index.

* Compared with septic shock group, *P* > .05.

** Compared with septic shock group, *P* < .05.

### 3.5. Laboratory indicators in peripheral blood of neonatal patients

Peripheral blood samples collected at admission revealed significant intergroup differences in white blood cell count (WBC) and creatine kinase-MB (CK-MB) levels (*P* < .05). No other laboratory parameters showed statistically significant variations. Details are presented in Table 5.

**Table 5:** Comparison of laboratory indicators in peripheral blood of neonatal patients.

Index	Septic shock group (n = 40)	Sepsis group (n = 42)	SIRS group (n = 74)	*F*	*P*-value
WBC (10^9^/L)	15.11 ± 7.68	15.88 ± 7.43*	12.62 ± 4.78*	3.851	.054
Neutrophils (%)	58.70 (48.98, 69.93)	68.25 (54.85, 78.83)*	52.85 (42.18, 66.25)*	8.423	<.001
C-reactive protein (mg/L)	15.59 ± 13.53	2.71 ± 5.96**	1.46 ± 4.89**	41.468	<.001
Interleukin-6 (ng/L)	198.92 ± 423.85	245.38 ± 501.86**	32. 26 ± 53.23**	6.291	.013
Procalcitonin (μg/L)	6.67 ± 21.63	1.77 ± 1.23**	0.52 ± 1.66**	7.301	.008
CK-MB (U/L)	26.51 ± 21.99	20.28 ± 13.39*	42.64 ± 205.46*	0.333	.565
NT-proBNP (μg/L)	7.90 ± 7.06	5.49 ± 5.33**	3.40 ± 5.32**	15.582	<.001
Lactate (mg/L)	4.95 (2.90, 7.65)	2.15 (1.38, 3.13)**	3.35 (2.30, 4.30)**	18.450	<.001
Urea (mmol/L)	3.63 ± 1.55	8.39 ± 18.41**	3.44 ± 1.29*	3.969	.021
Creatinine (μmol/L)	59.00 (50.00, 68.50)	58.00 (47.75, 67.00)*	51.00 (39.75, 58.25)**	13.004	<.001

Continuous data follows a normal distribution with (mean ± standard deviation), otherwise represented as median (interquartile range).

WBC = white blood cell count, CK-MB = creatine kinase isoenzyme MB, NT-proBNP = N-terminal B-type natriuretic peptide precursor.

* Compared with septic shock group, *P* > .05.

** Compared with septic shock group, *P* < .05.

### 3.6. Construction of a predictive model for septic shock

Variables with significant differences between the SIRS and sepsis groups were included in a logistic regression analysis to identify independent predictors of septic shock. Neonatal age, critical illness score, ScO_2_, PI of the RMCA, PSV of the LRH, procalcitonin, and lactate emerged as independent risk factors (*P* < .05). A multimodal predictive model was constructed using these variables:

Multimodal model = (−0.378 × neonatal age) − (0.145 × critical illness score) − (0.366 × ScO_2_) + (0.416 × PI of RMCA) + (0.825 × PSV of LRH) + (12.288 × procalcitonin) + (5.167 × lactate) + 1.804. Details are presented in Table 6.

**Table 6:** Multivariate logistic regression analysis of independent predictive factors for septic shock in neonatal patients.

Variables	β	SE	Wald χ^2^	OR	95% CI	*P*
Abort (yes = 1, no = 0)	1.038	5.558	3.425	2.816	1.964–8.676	.065
Preventive antibiotics (yes = 1, no = 0)	1.775	6.186	0.236	6.382	6.282–6.698	.326
Age of neonatal	−0.378	9.655	0.222	2.293	2.129–3.482	<.001
Birth weight	0.289	5.156	0.264	1.875	1.852–2.762	.264
Fetal distress (yes = 1, no = 0)	1.894	4.242	6.522	1.268	1.172–3.722	.357
First minute Apgar	−0.266	6.655	0.152	9.657	8.71–11.773	.982
Fifth minute Apgar	−1.845	3.316	0.422	2.321	2.095–12.895	.328
10th minute Apgar	−0.328	2.788	0.114	1.596	1.499–10.877	.342
Respiratory rate	0.452	7.47	8.629	2.972	2.284–3.289	.517
Systolic blood pressure	−0.173	8.643	8.958	0.789	0.725–1.888	.135
Diastolic blood pressure	−0.656	6.595	4.967	0.248	0.232–1.179	.175
Urine output	−1.639	2.626	0.296	0.562	0.533–0.788	.837
SPR (yes = 1, no = 0)	1.858	1.838	0.223	0.853	0.827–1.322	.462
Dopamine (yes = 1, no = 0)	−0.204	3.611	0.521	0.154	0.132–0.959	.703
Critical illness score	−0.145	4.352	0.875	1.835	1.582–2.354	.038
PtcO_2_	0.552	4.492	0.106	0.962	0.921–2.055	.994
ScO_2_	−0.366	1.652	0.123	0.289	0.281–0.359	.021
rSO_2_	–1.828	2.259	5.758	1.526	1.444–1.595	.583
PI of RMCA	0.416	0.22	0.569	0.837	0.828–1.022	<.001
PSV of LRH	0.825	7.556	0.187	0.952	0.868–1.157	.001
RI of LRH	0.789	2.264	0.316	0.462	0.452–0.672	.482
C-reactive protein	1.875	3.756	0.158	2.961	2.351–9.898	.508
Interleukin-6	0.392	6.922	0.069	0.203	0.189–0.577	.525
Procalcitonin	12.288	1.642	0.265	1.875	1.725–2.061	.004
NT-proBNP	1.896	2.438	6.522	1.262	1.227–3.727	.357
Lactate	5.167	6.526	0.151	9.654	8.612–10.572	.013
Constant value	1.804	3.112	0.422	2.329	1.925–2.897	.002

LRH = left renal hilum, NT-proBNP = N-terminal B-type natriuretic peptide precursor, PI = pulsatility index, PSV = peak velocity, PtcO_2_ = transcutaneous oxygen pressure, RI = resistance index, RMCA = right middle cerebral artery, ScO_2_ = cerebral oxygen saturation, SPR = sensitive pupil response.

### 3.7. Predictive value of multimodal predictive models in predicting septic shock

**Figure 1.**
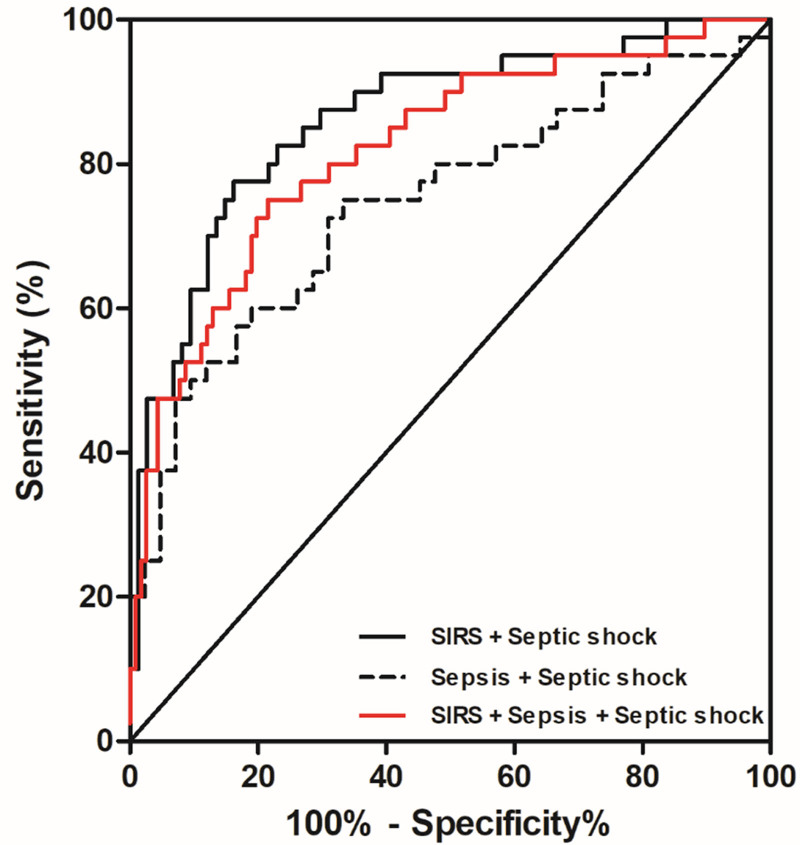
ROC curve of predicting septic shock occurrence in different populations based on multimodal data prediction model. ROC = receiver operating characteristic curve.

ROC curve analysis demonstrated the model’s discriminatory power. The area under the curve for predicting septic shock was 0.862 in the SIRS + septic shock cohort, 0.746 in the sepsis + septic shock cohort, and 0.820 in the combined SIRS + sepsis + septic shock cohort. Sensitivity and specificity were 83.78% and 77.50%, 66.67% and 75.00%, and 78.45% and 75.00%, respectively. Details were presented in Table 7 and Figure 1.

**Table 7:** Predictive value of a predictive model based on multimodal data in predicting the occurrence of septic shock in different populations.

Populations	AUC	95% CI	*P*	Specificity (%)	Sensitivity (%)
SIR + septic shock	0.862	0.789–0.935	<.001	83.78	77.50
Sepsis + septic shock	0.746	0.638–0.854	<.001	66.67	75.00
SIR + sepsis + septic shock	0.820	0.741–2.899	<.001	78.45	75.00

AUC = area under the receiver operating characteristic curve, CI = confidence interval, SIR = systemic inflammatory response.

## 4. Discussion

Neonatal infections represent a leading cause of morbidity, mortality, and healthcare resource utilization worldwide, with marked regional disparities. Notably, economically developed regions exhibit lower incidence rates. Severe infections can trigger host dysregulation, culminating in life-threatening organ dysfunction, termed sepsis, which may progress to septic shock.^[3]^ In China, the incidence of neonatal sepsis among survivors ranges from 4.5‰ to 9.7‰, yet mortality rates remain alarmingly high (30%–50%), with refractory shock or multiple organ dysfunction syndrome emerging as the predominant causes of death, typically within 48 to 72 hours of symptom onset.^[14,15]^ This retrospective study enrolled 156 neonates admitted with suspected infection, of whom 74 were diagnosed with systemic inflammatory response syndrome (SIRS), 42 with sepsis, and 40 with septic shock. Given the substantially higher mortality associated with septic shock compared to non-shock sepsis or SIRS, early differentiation among these conditions is critical. To address this, we comprehensively analyzed clinical data collected at admission, including maternal history, baseline patient characteristics, imaging findings, noninvasive hemodynamic parameters, and initial laboratory results. These variables were compared across diagnostic groups.

Univariate and multivariate logistic regression analyses identified 7 independent predictors of septic shock: neonatal age, critical illness score, ScO_2_, RMCA, PSV of LRH, procalcitonin, and lactate levels. Notably, 37/40 (92.5%) neonates with septic shock were admitted within 1 hour of birth, compared to 15/42 (35.7%) in the sepsis group and 39/74 (52.7%) in the SIRS group. This suggests that intrauterine infections may predominate in septic shock cases, potentially due to prolonged pathogen exposure and immature immune defenses, leading to rapid clinical deterioration.^[16,17]^ The neonatal critical illness score, a validated tool for assessing disease severity,^[18,19]^ demonstrated prognostic utility, as higher scores correlate with increased mortality.^[20,21]^

The pathophysiology of shock involves tissue hypoxia from impaired organ perfusion, ultimately progressing to multi-organ failure (e.g., brain, kidneys, liver).^[22,23]^ Cerebral and renal dysfunction are particularly critical in sepsis-related mortality.^[24,25]^ Among the predictors identified, ScO_2_ and RMCA-PI reflect cerebral perfusion, while LRH-PSV correlates with renal perfusion. Near-infrared spectroscopy-derived ScO_2_ provides continuous, noninvasive monitoring of cerebral oxygenation and blood volume, enabling early detection of hypoperfusion and timely intervention to mitigate neurological injury.^[26–28]^ In this study, we used near-infrared spectroscopy to measure the concentration of oxyhemoglobin in brain tissue, i.e., ScO_2_, which mainly reflects the blood oxygen content of cerebral veins, and can continuously and directly and noninvasively monitor cerebral oxygen metabolism status and cerebral blood volume.^[29,30]^ In recent years, it has been reported that monitoring the change of PI and PSV value can accurately reflect the level of tissue perfusion.^[31,32]^ Compared with the monitoring of central venous pressure, it has the advantage of noninvasive continuous evaluation, and is more accurate than the peripheral filling time to judge the tissue perfusion level, so the monitoring of PI and PSV value is of great significance for early diagnosis of perfusion disorders and avoiding tissue hypoxia and organ failure.^[31,32]^

Procalcitonin and lactate are endorsed by international sepsis guidelines as pivotal diagnostic and prognostic biomarkers.^[3,33]^ Procalcitonin, a calcitonin precursor undetectable in healthy individuals, rises rapidly during bacterial infections, with levels proportional to disease severity.^[34,35]^ Its diagnostic performance surpasses conventional markers like C-reactive protein,^[36,37]^ and serial measurements guide antibiotic stewardship.^[38,39]^ Blood lactate level is an important indicator reflecting tissue perfusion and cellular hypoxia. Children with sepsis exhibit varying degrees of tissue hypoperfusion and oxygenation disorders. These conditions lead to anaerobic metabolism, resulting in excessive lactate production.^[40–42]^

Using these predictors, we developed a multimodal predictive model for neonatal septic shock. The model demonstrated robust discriminative ability, with areas under the curve of 0.862 (SIRS + septic shock), 0.746 (sepsis + septic shock), and 0.820 (combined cohort), indicating strong clinical utility.

## 5. Conclusions

This study identified key predictors of neonatal septic shock and established a multimodal predictive model with promising diagnostic accuracy. Nevertheless, several limitations warrant consideration. First, the retrospective single-center design introduces potential selection bias. Second, the modest sample size necessitates validation through multicenter studies with larger cohorts. Future research should also explore dynamic biomarker trends and integrate novel technologies to refine risk prediction.

## Acknowledgments

Thanks to the following funds for funding this study: the Natural Science Foundation of Fujian Province (2022J011296), Fujian Provincial Department of Science and Technology, Fuzhou Neonatal Key Specialty Project (20191204), and Fuzhou First General Hospital Science and Technology Project (2022-YJ-07).

## Author contributions

**Conceptualization:** Fan Liu, Zheng Yan.

**Data curation:** Jinlan Chen.

**Formal analysis:** Qinglan Huang, Xiaoning Yan.

**Funding acquisition:** Fan Liu.

**Investigation:** Xiaoning Yan.

**Methodology:** Qinglan Huang, Xiaoning Yan.

**Resources:** Fan Liu.

**Software:** Jinlan Chen, Zhen Li.

**Supervision:** Zheng Yan.

**Validation:** Zheng Yan.

**Visualization:** Zheng Yan.

**Writing – original draft:** Fan Liu.

**Writing – review & editing:** Zheng Yan.
